# Heads and Tails: An Analysis of Visual Signals in Cats, *Felis catus*

**DOI:** 10.3390/ani11092752

**Published:** 2021-09-21

**Authors:** Bertrand L. Deputte, Estelle Jumelet, Caroline Gilbert, Emmanuelle Titeux

**Affiliations:** Ecole Nationale Vétérinaire d’Alfort, 7 Avenue du Général de Gaulle, 94704 Maisons-Alfort, France; estelle.jumelet@gmail.com (E.J.); caroline.gilbert@vet-alfort.fr (C.G.); emmanuelle.titeux@vet-alfort.fr (E.T.)

**Keywords:** visual communication, cats, *Felis catus*, cat–cat interactions, cat–human interactions, welfare

## Abstract

**Simple Summary:**

Communication between individuals of the same species is essential in their interactions to regulate their proximities and distances. Communication includes exchanges of more or less complex visual signals. We attempt to decipher the most significant features of a visual configuration involving the combination of tail and ear positions in interactions between cats. Although the tail is a conspicuous feature for human observers, we demonstrate that ear positions of the cats in dyadic interactions with other cats are the best predictor of the outcomes of these interactions. However, in cat–human interactions, the cat most often approached with its tail up prior to rubbing itself against the human. The results are important for a better understanding of cats’ perceptions of humans, and will help to promote cat welfare.

**Abstract:**

Visual communication involves specific signals. These include the different positions of mobile body elements. We analyzed visual configurations in cats that involve ears and the tail. We aimed at deciphering which features of these configurations were the most important in cats’ interactions with other cats and with humans. We observed a total of 254 cat–cat interactions within a sample of 29 cats, during a total of 100 h of observation scheduled with the “Behavioral dependent onset of sampling” method and using the “All occurences” sampling method. In addition, we sampled 10 interactions between cats and humans. In cat–cat interactions, we noted the positions of ears and tail of both protagonists, as well as the outcome of the interaction, which was either positive/neutral or negative. In a great majority of the 254 interactions sampled, both cats held their tail down. On the contrary, ear position was a critical element in predicting the outcome. When both partners held their ears erect, the outcome was significantly positive, such as rubbing or close proximity. In all other cases of the position of ears in both cats, the outcome was negative, with increased distance of the partners. Although the tail did not seem to play a significant role in visual configurations in cat interactions, the “tail-up” display was important when a cat approached a human being. In the vast majority of cases the cat rubbed itself on a human’s leg(s). Thus, we may conclude that the presence of a human has a specific meaning in the cat’s world, probably as the result of a long period of commensalism. It is important for pet owners to understand the signals that cats use with other cats and with humans in order to promote the welfare of cats.

## 1. Introduction

Social interactions involve the process of communication. As Marler [1] pointed out, communication leads to the regulation of proximities and distances between individuals of any species, though especially in social species, such as most primates. In the course of a dyadic interaction, a sender emits signals through one or more channels to a receiver. The receiver decodes these signals. However, the receiver also processes the information based on the sender or modified by context. While it is possible for an observer to determine characteristics of the sender (its sex, size, etc.), it is not possible to reliably know what cues from the context have been processed by the receiver. Menzel and Johnson [2] considered context to be similar to a “garbage pail”, as it is not possible to assess precisely which information is processed among a large quantity of information of different kinds.

In every species, signals have been shaped during evolution through different processes, identified by Tinbergen and grouped under the concept of “ritualization” [3]. These processes include, for example, “emancipation” from the original physiological or other function, and “stereotypy” that reduces the variability of the expression of future signals.

Visual signals are controlled by specific motor patterns that concern mobile parts of the body. Visual signals are often referred to as “displays”. Displays include all visual patterns that can be perceived by other conspecifics and possibly lead to changes in their behavior. Displays, in reality, represent complex patterns of signals [4]. Here we label “configurations” as a different kind of visual pattern, involving postures and different positions of mobile elements such as ears, tail, mouth, etc. In contrast to the holistic aspects of displays, “configurations” might be considered as combinations of clearly identified movements of mobile parts of an animal’s body, including its head. As a consequence, specific configurations could be analyzed based on their components. Configurations correspond to the referentials that Golani [5] proposed to describe movements involved in mammalian interactions, particularly, the self-referential “bodywise” and the other-referential “partnerwise”. As far as the “bodywise” referential is concerned, the different mobile parts of the body of an individual (head, mouth, ears, neck, back, etc.) may move with respect to other mobile parts. The “partnerwise” referential refers to the relative positions of the protagonists of a dyadic interaction. The description of an interaction must therefore consider simultaneously two referentials, the “bodywise” one and the “partnerwise” one. In some mammal species, the tail is an important element included in the “bodywise” referential. Very often the position of the tail and the facial expressions are analyzed separately. However, all these movements should be considered together as yielding particular “configurations” within the “bodywise” referential. The question arising from the simultaneous occurrences of different positions of some mobile body elements, might be, for example: “Does the meaning of an individual approaching another one with a certain facial expressions with the tail between its legs similar to this individual approaching its partner with the same facial expressions but with it tail held up”? When facial expressions are analyzed separately from tail positions and vice-versa, it is impossible to answer this question. This issue of the complexity of expressions of emotions is not restricted to “unimodal” configurations, such as the visual ones previously mentioned. It also applies to “bimodal” configurations, especially “visual-vocal” configurations. In general, vocalizations are thoroughly analyzed, but separately from different postures or facial expressions. The demeanor of the sender is often mentioned but not specifically analyzed along with the vocalizations, e.g., as in [6]. Partan and Marler [7] addressed the issue of the multimodality involved in communication events and questioned whether a unimodal signal had the same meaning when it was sent alone as opposed to in combination with a signal from another modality. Baraud et al. [8] demonstrated that, when associated with different postures or other visual behaviors, vocalizations, instead of providing redundant information, provided additional information that appeared to reduce the possible ambiguity of the visual signal alone. As far as visual signals are concerned, Kiley-Worthington [9] explored in many mammalian species the potential function of tail position in interactions alongside other motor patterns. She suggested that the use of the tail in displays is connected to its primary function as an organ of locomotion. It appears to her, therefore, that tail movements in displays are not emancipated from their original causation [9]. There have been mixed results in empirical studies. In baboons, Hausfater [10] showed that a neutral tail carriage could not be correlated with dominance rank in males, as it was in females, and reported no sex differences in the use of tail position. In addition, neutral tail carriage of first-ranking males and females did not differ from that of other individuals. Hausfater [10] therefore concluded that in baboons, tail carriage does not serve as a signal linked to change in social status, but merely morphological changes. Schenkel [11] reported different positions of the tail in wolves. He assigned moods to each of them, but only on the basis of subjective observations. In addition, the tail, whatever its position relative to the body, may be still or waving at different amplitudes and frequencies. In wolves, Harrington and Asa [12] demonstrated that tail base position was related to social status. This claim has been confirmed in dogs [13]. However, further experimental work is needed to evaluate the communicative role of tails.

Whereas vocalizations can be thoroughly analyzed and dissected into a number of discrete variables (pitch, frequency modulation, loudness, and structure, such as tonal vs. atonal), visual displays are rarely broken down into their components. One significant exception might be the Facial Action Coding System, proposed by Ekman [14] to analyze facial expressions in humans. Ekman’s system has been adapted to several species for different purposes (e.g., chimpanzees [15], MaqFACS in macaques [16]), especially for the assessment of pain (horses [17] and laboratory mice [18,19]). The position of ears, as part of configurations including facial expressions, is an important visual signal in some domestic species such as dogs and horses, [20,21], respectively).

Cats are usually considered as a solitary species, though cats may gather themselves in groups centered on food resources [22,23,24,25]. Cats, as a species, are non-social and non-solitary. Cats show a highly flexible inter-individual tolerance, unusual in a solitary species (e.g., [26]). Darwin [27] gave a vivid, detailed description of aspects of cats in different contexts as expressing different emotions. Darwin’s description involved the general posture of the cat, the position of ears, the opening of the mouth, the position of the tail, and its possible very varied movements. “Configurations” in cats might be very easily drawn from these descriptions. Later, Leyhausen [23] illustrated the principal facial expressions of cats along two postulated motivational dimensions, aggression and fear. Leyhausen’s drawings exemplified what we refer to here as “visual configurations”. The component of cats’ facial “configurations” involve, in particular, ear movements, opening of the eyes, and opening of the mouth, generally associated with vocalizing ([23], p. 195). Since this original work, several studies have provided support to some of the facial expressions presented by Leyhausen, especially in applying the FACS concept to cats (CatFACS) in order to assess different aspects of cat behavior in experimental conditions [28,29,30,31]. Bennett et al. [28], using CatFACS and a cluster analysis, provided an experimental quantitative support to some facial expressions initially presented by Leyhausen [23]. Their experimental settings allowed them to precisely associate specific emotions to facial expressions.

Along the same aggression/flight dimensions, Leyhausen [23] illustrated bodily “configurations”. These include positions of the body in relation to the ground, the line of the back, and the position of the tail ([23], p. 194). Bradshaw and Cameron-Beaumont reported ear postures in cats [32] (p. 73). It is worth noting that more than ear postures are featured in that figure. The figure actually features what we call “visual configuration”, as posture, head and neck, mouth, and eyes are clearly differentiated. Bradshaw and Cameron-Beaumont [32] analyzed what they referred to as the “Tail up display”, a specific erect position of the tail during cats’ interactions. They considered whether the interacting individuals, sender and/or receiver, held their tails up or not. Cafazzo and Natoli [33] analyzed the use of the tail-up display in a colony of cats in Rome. They found that the tail-up display was a friendly signal. In addition, the tail-up display was more frequent in females and more frequently associated with subsequent rubbing than with subsequent nose sniffing. They reported that the tail-up display was more often displayed by low-ranking cats than high-ranking ones. They also reported that their dominance hierarchy, based on aggression given and aggression received, was highly correlated with age. So, according to their analysis, the oldest cats aggressed the most toward the youngest ones. The latter, when approaching an older cat, held their tail up, likely to inhibit expected aggression based on previous encounters.

In all these studies, ear positions or tail position were studied separately (see also [34]). In addition, Cameron-Beaumont [35] analyzed separately either the sender’s tail position or that of the receiver. 

However, during interactions, individuals continuously exchanged signals and processed information in a manner that Tinbergen [4] referred to as a “chain of reactions”, which he illustrated with the zig-zag dance of courtship in sticklebacks [36]. As a consequence of the nature of interactions, one might argue that sender’s and receiver’s signals were not independent nor independent of the other components of “holistic” visual signals. 

This study is aimed at analyzing “visual holistic configurations” instead of considering their elements separately. This study aimed at deciphering which components of visual configurations are the most important in cat interactions. We focused here on the position of the ears and that of the tail. In addition, we compared these visual configurations during cat–cat versus cat–human interactions.

## 2. Housing, Subjects, and Methods

### 2.1. Housing and Subjects

**Figure 1:**
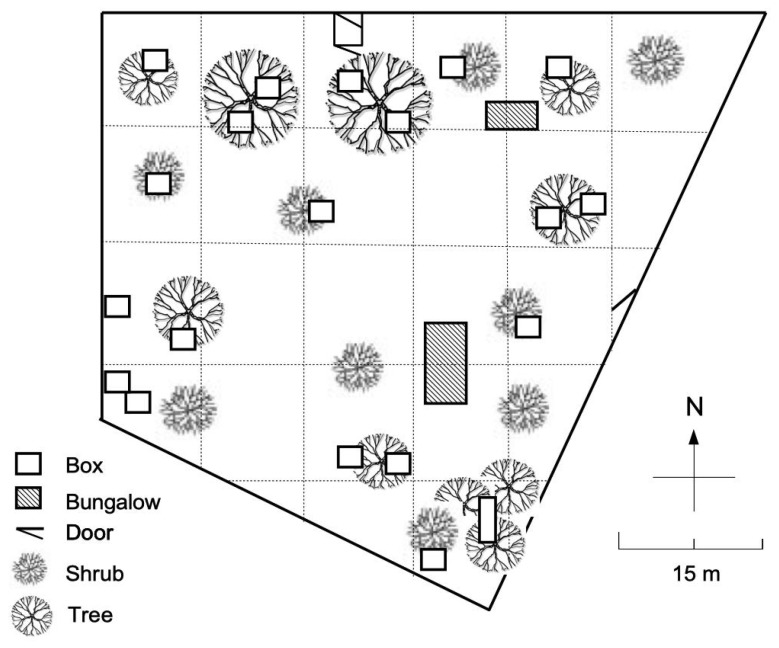
Schematic map of the AVA enclosure. Its area was about 2600 m^2^, which housed a captive population of 40 to 50 cats. The enclosure included grass, trees, bushes, shrubs, litter boxes, and suitable places for micturition and defecation, fallen trees and other places for scratching, boxes that could house one or two cats, and bungalows that can be heated and that were equipped with beds, blankets, and could house a score of cats.

The study was conducted in a colony of cats in a shelter (AVA, Cuy Saint Fiacre, France). The AVA shelter housed cats that were stray cats, abandoned pet cats, or pet cats left to the shelter by their owners. The area of the enclosure was about 2600 square meters. The enclosure included grass, trees, bushes, shrubs, litter boxes, and suitable places for micturition and defecation, fallen trees and other places for scratching, boxes that could house one or two cats, and bungalows (Figure 1). Bungalows could be heated and were equipped with shelves, beds, blankets, and could house a score of cats. Feeding and drinking places were available at different spots near the bungalows.

#### 2.1.1. Ethical Note

AVA means “Aide aux vieux Animaux” (“*Help to Old Animals*”). With this being the case, cats who are living at the shelter are intended to be adopted as pet cats If not adopted after entering the shelter, they will spend the rest of their lives at the shelter. A trained staff, including two veterinarians, takes care of the cats. All subjects were free from FIV and FeLV. The AVA Association which runs the facilities complies with the French regulations. The Director approved our non-invasive observational study.

#### 2.1.2. Subjects

A group of around 50 cats were living in the 2600 m^2^ enclosure. Therefore, cats could easily regulate their distance towards other cats in the colony, seeking proximity and contact or increasing interindividual distance. During a familiarization phase prior to the quantified observations, the observer (EJ) identified twenty-nine cats among the population of 40 to 50 cats. Identification was based on coat color and patterns and other cues if necessary. The 29 identified cats were those that were the most visible any time in the enclosure. As cats were intended to be adopted, the population might vary accordingly. Identified cats remained within the enclosure for the duration of the study. As cats were free to range within the enclosure they were not manipulated routinely by the staff. The caregivers entered the enclosure twice a day for feeding and cleaning the bungalows and boxes. As cats might remain hidden within the enclosure, the caregivers interacted only with cats who approached them. Cats were never forced to be manipulated. The identified cats included 13 males, 5 females. For 11 cats the sex could not be determined. Cats were all cross-breed cats. They were either neutered [11 cats], intact [4 cats], or of unknown status [14 cats]. The information about the sex and reproductive status of the cats was provided by the staff. Cats of the enclosure were moderately attracted to humans, although familiar with them. So the presence of the observer within the enclosure did not modify the behavior of the cats and did not prevent cat–cat interactions. 

### 2.2. Methods

#### 2.2.1. Observations

Observations were completed during 2 periods, in winter and summer—three 5-day weeks in February—two 5-day weeks early September 2006. Sampling took place at 4 different times a day (8 h, 12 h, 14 h, and 18 h) for 1 h each. A total of 100 h of observation was completed. Prior to observations, a map of the enclosure was drawn. The map displayed all the different elements that existed within the enclosure (Figure 1). On the map the enclosure was divided into virtual 20 m × 20 m quadrats as far as the geometry of the enclosure allowed (Figure 1). These quadrats helped to precisely localize the cats within the quadrats and in respect to the specific structures or elements of the enclosure. During an observation session, the observer (EJ) positioned herself at different places of the enclosure to sequentially cover, visually, the whole enclosure. The order in which the different spots were used varied randomly within daily observations and between days of observations. During stops at different places, the observer scanned the environment around her and started the observation sessions using the “Behavior dependent onset of sampling” [37]. The dependent behavior that marked the onset of the sampling was an approach: an identified cat walked towards another identified cat with its head and body oriented to the other cat. To assess, one cat approached another; the initial interindividual distance never exceeded 3 m. The cat which walked towards another one is referred to as the “Initiator” of the interaction, the other cat was considered as the “Receiver”. Our sampling also included approaches to humans, although only the behavior of the “initiator” cat was considered. During observation sessions, when approached by a cat, humans had to stand still and were instructed to not interact with the cat. Humans approaching cats were discarded from our sampling. During interactions the “All occurrences” sampling method was used [37]. At the different observation spots, the observer remained still, and at a distance from the cats that varied from less than one meter to several meters. The observer never interacted with the cats. Only interactions—either cat–cat or cat–human—that involved identified cats were taken into account.

The sampling resumed as soon as both cats moved away from each other, or only one cat moved away, or both remained in contact or close proximity. The outcome of the interaction was referred to as a “negative” whenever it might be considered as agonistic, involving aggressive behaviors or/and flight or avoiding behaviors. The outcome of an interaction was considered as “positive-friendly” or “neutral” whenever the two cats remained in contact, sniffing noses or engaging in mutual rubbing or licking, remained sitting or lying in close proximity, or when one of the cats simply passed on. Therefore, the outcome of each interaction depended on the variation in interindividual distance, which initially decreased as the consequence of the approach (c.f. [1]).

All interactions were noted by hand. A total of 254 interactions involving identified cats was sampled. In addition, 104 cat–human interactions were noted.

#### 2.2.2. Variables

At the onset of the interactions, the position of the tail and the position of the ears were noted for both the initiator and the receiver cats. The position of the tail and that of the ears were considered simultaneously in both cats.

We defined these combinations of body elements as “visual configurations”, which corresponded essentially to the “bodywise” referential defined by Golani [5]. Three modalities of ear positions were considered: “erect”(c.f. position A_0_B_0_ and A_1_B_0_ [23] (p. 195), see also “forward and erect” [32] (p. 73) and [28]), “flattened” on the sides of the head (c.f. position A_0_B_1_ and A_1_B_2_ [23] (p. 195), “flat” [32], and see [28]), and “moved down and backwards” (c.f. position A_0_B_2_ [23] (p. 195), “back and flat”, [32], and see [28,34]). Two modalities of tail position were considered: “Up”, as defined in Cameron-Beaumont’s study [35], and whether the tail was held horizontal or below the plane of the cat’s back. In both modalities the possible movements of the tail were not taken into account.

#### 2.2.3. Coding Methodology

**Figure 2:**
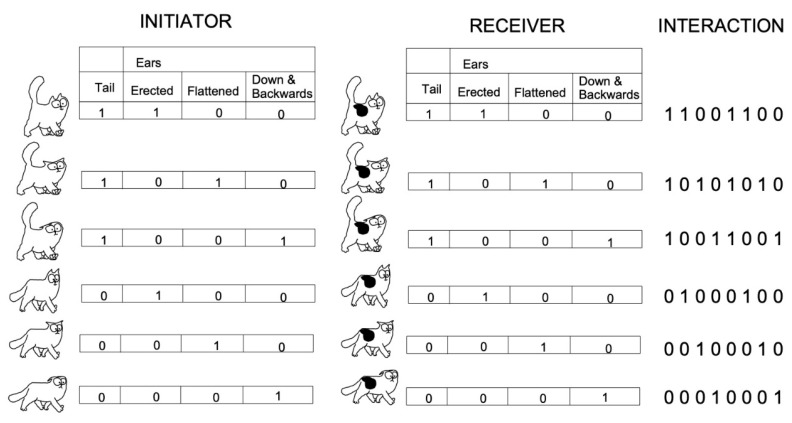
Coding system of the visual configurations in cats’ interactions. The configurations only involved the position of the tail and that of the ears in each partner. The position of the tail is coded by one digit—1, tail up, and 0, tail down. The positions of the ears are coded on 3 binary digits depending on the 3 considered positions. Therefore, for each cat the 6 visual configurations are coded on 4 binary digits. The dyadic initiator (I)–receiver (R) interactions are then coded on 8 binary digits. Interactions included 36 combinations of IR visual configurations.

Our goal was to take into account simultaneously the configurations made by the positions of ears and tail in both cats. For each ear position, there could be two different positions of the tail, “up” or “not” (Figure 2). The coding for each cat is a 4-digit number based on a binary system where the tail position is coded 1 for “Up” or 0 for “Not up” and the position of the ears on 3 digits with 1 for the position observed and 0 for the 2 other modalities (Figure 2). 

The outcome, “positive/neutral” or “negative”, was assigned to each coded interaction. The coding system was also applied to cat–human interactions, though only for the cat. So in that case the interaction was only coded by a 4-digit number.

### 2.3. Data Processing and Analyses

The G-test was used for goodness-of-fit tests (even distribution) and for independence (association) in one-way RxC contingency tables [38]. Observed G values were compared to Chi^2^ values for particular degrees of freedom. The Chi^2^ and the binomial tests were also used as goodness-of-fit tests or for contingency tables, depending on whether they were applied to independent samples or one-sample cases [38,39]. Testing the equality of two percentages was used as well [40]. All tests were two tailed and the critical *p* was set at 0.05. 

## 3. Results

### 3.1. Configurations Expressed during Cat–Cat Interactions

**Figure 3:**
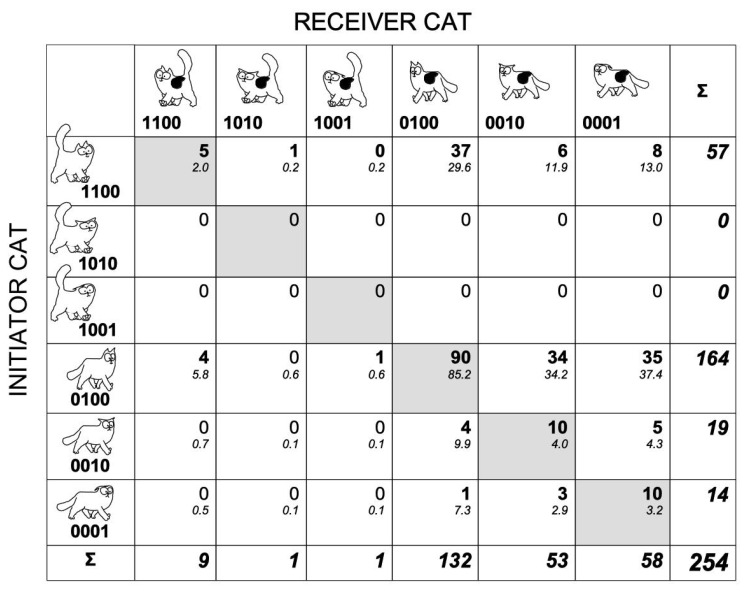
Matrix of tail–ears configurations in cat dyadic interactions. The 6 × 6 matrix of tail–ears configurations in the two individuals of 254 dyadic interactions. The rows feature the 6 possible configurations in the initiator cat, and the columns feature the 6 possible configurations in the receiver cat. The cells of the matrix included the frequencies of the combinations of I and R configurations. The observed frequencies are indicated in bold numbers. The expected frequency for a cell, i,j, is computed as the total of the column, j, times the total of the line, i, divided by the grand total. The expected frequencies were featured in lower italic numbers. The last row and column featured the sum of the frequencies for the receiver and the initiator, respectively. The cells along the diagonal of the matrix are in light gray and feature the configurations which are similar in both cats. As only 16 IR configurations have been observed, the statistical analyses required the pooling of some configurations or the use of a reduced matrix.

We set up a 6 × 6 contingency table, where the rows represent the initiator’s configurations (combinations of tail and ear positions) and the columns the receiver’s configurations. The cells represent the frequency of different interactions. The diagonal of the table included the frequencies of the configurations similar to both cats, initiator and receiver. Among the 36 possible configurations, less than half of them (16: 44.4%) were actually observed (Figure 3). The initiator cat approached the other cat with the tail down in 77.6% of the interactions (197 out of 254 interactions; Figure 3). 

**Figure 4:**
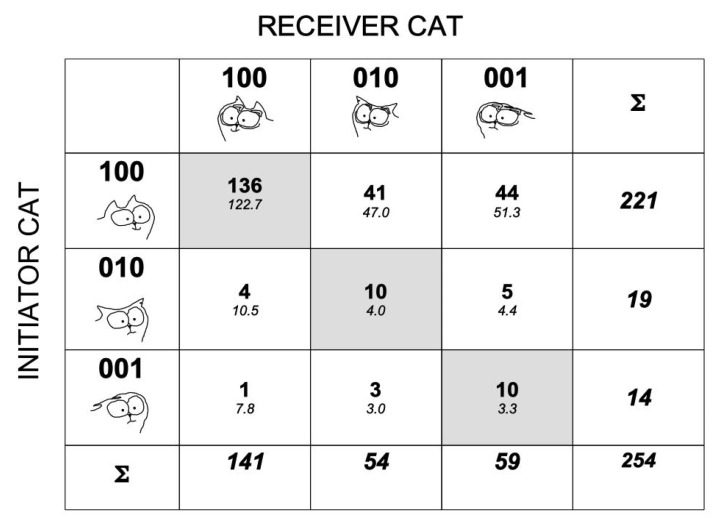
Matrix of ear positions in cat dyadic interactions. The 3 × 3 matrix of positions of the ears in the two cats in 254 dyadic interactions. The rows feature the 3 possible positions in the initiator cat; the columns feature the 3 possible ones in the receiver cat. The cells of the matrix included the frequencies of the combinations of I’s and R’s ear positions. The observed frequencies are shown in bold numbers. The expected frequencies are shown in lower italic numbers (see Figure 2 for the computation of these expected frequencies). The last row and column featured the sum of the frequencies for the receiver and the initiator, respectively. The cells along the diagonal of the matrix were colored in light gray. They featured ear positions where they were similar in the 2 cats. As some expected frequencies were quite low, the statistical analyses required the pooling of some ear positions to be completed.

In 75.6% of the interactions both cats showed a tail-down display (192 out of 254 interactions). Both cats interacted with their tail up whatever their respective positions of their ears, in only 2.4% of the interactions (6 out of the 254 tail–ears configurations, Figure 3). The initiator displayed a tail-up display while approaching the other cat, whatever the recipient’s tail–ears configuration, in 22.4% of the interactions (57 out of 254). Consequently, it was not possible to compute a G-test of association on all tail–ears configurations observed even when pooling suitable rows and/or columns. When considering only the configuration where the initiator cat approached with the tail up and ears erect (code 1100), the corresponding configurations in the receiver were not distributed evenly (first line of contingency table; G = 81.4, df = 5, *p* < 0.001; Figure 3). The configuration of the receiver cat where it had its tail down and its ears erect was observed significantly more often than expected (Chi^2^ = 79.6, df = 1, *p* < 0.001; Figure 3). There was a trend that both cats showed the same tail–ears configuration (diagonal of the table, 45.3% of observed cases for only 6 out 36 possible configurations (16.7%; Figure 3)). Since in the majority of the interactions both cats held their tail down, it is likely that it is the positions of the ears that convey most of the information concerning cat emotional states. After this, we created a 3 × 3 contingency table with the ear position of the initiator cat as rows and those of the receiver cat as columns of the table (Figure 4). 

There was a significant association between the ear position displayed by the initiator and the receiver cat (G = 27.8, df = 2, *p* < 0.001; Figure 4). Both cats had the same ear position during an interaction when the initiator cat did not have its ears erect significantly more often than expected (Figure 4).

### 3.2. Tail–Ears Configurations at the Onset of Interactions and Outcomes of These Interactions

**Figure 5:**
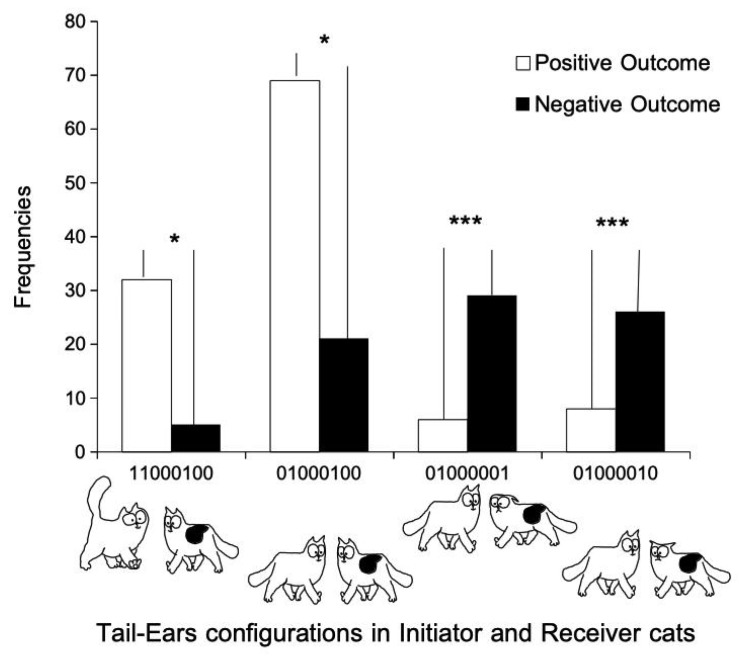
Outcomes of interactions depending on I–R tail–ears configurations (*n* = 196). Only 4 tail–ears configurations could have been analyzed. * *p* < 0.05; *** *p* < 0.001.

The outcomes of only 196 interactions have been assessed. As some configurations were rare, we had to pool some configurations to obtain large enough frequencies. As a consequence, only four tail–ears configurations were analyzed, with only one where the initiator had its tail up. We then constructed a 4 × 2 contingency table, featuring four initiator–receiver tail–ears configurations and two modalities of outcomes, positive and negative (Figure 5).

There was a significant association between some configurations and outcomes (G-test, G = 52.14, df = 3, *p* < 0.001). When both cats had their ears erect, the receiver its tail down, and regardless of the tail position of the initiator, the outcomes were positive significantly more often (Chi^2^ = 5, df = 1, *p* < 0.05 in both cases, Figure 5). On the contrary, when the cat initiating the interaction had its tail down and its ears erect, and the receiver also had its tail down and its ears non-erect, the outcomes were negative significantly more often (receiver ears flattened: Chi^2^ = 14.6, df = 1; backwards: Chi^2^ = 10.2, df = 1; both cases *p* < 0.0001; Figure 5).

**Figure 6:**
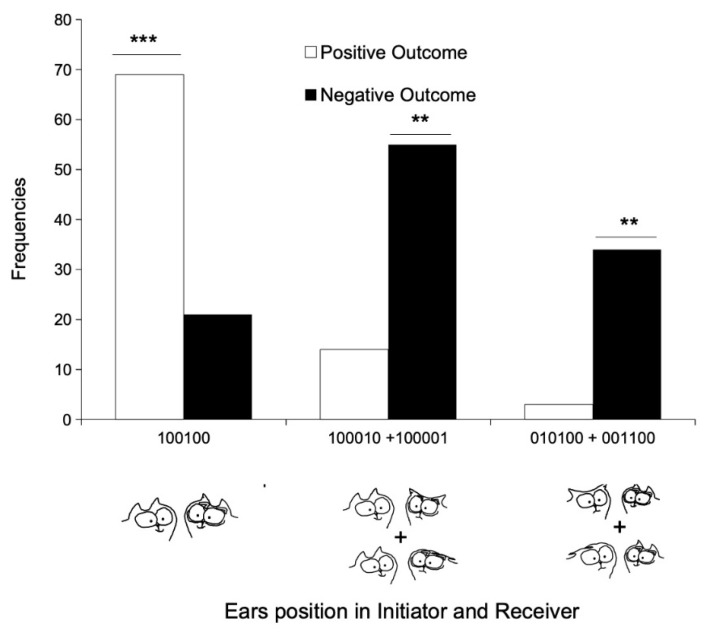
Outcomes of interactions depending on I–R ear positions (*n* = 196). Only 3 I–R ear positions occurred frequently enough to be analyzed. Two of the three categories included pooling of different ear positions. In the first 2 categories, the initiator cat had its ears erect. In the 3rd category the receiver cat had its ears erect. ** *p* < 0.01; *** *p* < 0.001.

To consider only the ears position regardless of associated tail position in both cats, we constructed a 4 × 2 contingency table that included the following interactions: one—ears erect in both partners (100100), two—ears erect in the initiator vs. ears non-erect in the receiver (ears flattened and ears backwards—100010 plus 100001), three–ears non-erect in the initiator and erect in the receiver (010100 plus 001100), and four—ears non-erect in both partners (010010 plus 010001 plus 001010 plus 001001). Due to low frequencies, we had to pool the last two categories (initiator ears non-erect and receiver either ears erect or not, respectively, 010100 plus 001100 plus 010001 plus 001010), leading to a 3 × 2 table, with three ear configurations (ears erect in both partners, ears erect in initiator and non-erect in receiver, and ears non-erect in both partners, with two modalities of outcomes, positive and negative (Figure 6). 

Out of the 196 interactions observed, about half of them were either positive (43.9%) or negative (56.1%). There was a significant association between ear configuration in both partners and the outcomes of the interaction (G = 80.55, df = 2, *p* < 0.001; Figure 6). When both cats interacted with their ears erect, the outcomes were significantly likely to be positive (Chi^2^ = 22.1, df = 1, *p* < 0.0001; Figure 6). The interaction then led to prolonged contact, rubbing, licking, or close proximity. On the contrary, when both cats held their ears in different positions or had their ears non-erect, negative outcomes, such as flight, avoidance, or frozen defensive posture were significantly more probable than expected (erect vs. non-erect or non-erect vs. erect plus non-erect, Chi^2^ = 6.8, df = 1 and Chi^2^ = 8.4, df = 1, respectively; both *p* < 0.01; Figure 6). Most of the negative outcomes occurred when one of the cats, especially the receiver, or both cats had their ears non-erect (80.9% of the negative interactions; Figure 6).

### 3.3. Tail Position According to Intra- and Interspecific Approaches

In the large majority of cat–human interactions, cats approached the humans with their tail up and ears erect (code 1100 plus code 1001, 97.8%). When approaching with their tail down, cats always held their ears non-erect, either flattened or backwards.

**Figure 7:**
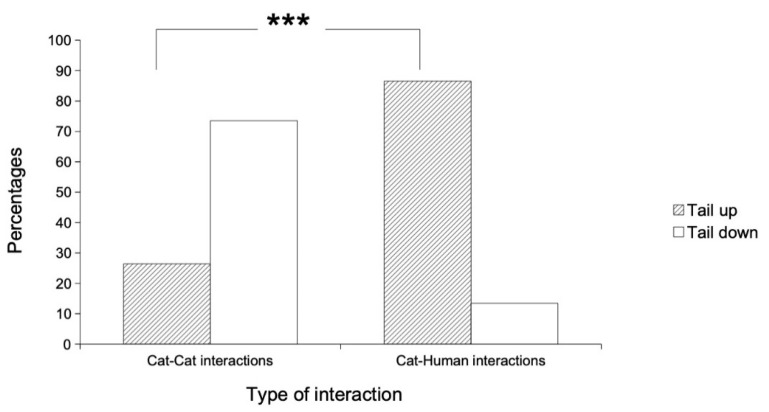
Tail positions depending on either cat–cat or cat–human interactions. The tail-up position was observed significantly more often in cat–human interactions than in cat–cat interactions. *** indicated a significant difference with *p* < 0.001. Cat–cat interactions, *n* = 257; Cat–human interactions, *n* = 104.

We compared the tail positions of the cats that were sampled in 257 cat–cat and 104 cat–human interactions (2 × 2 contingency table, Figure 7). 

The tail position was significantly associated with a certain type of interaction (G = 115.7, df = 1, *p* < 0.0001; Figure 7); the tail-up display in cat–human interactions was observed significantly more than expected (Chi^2^ = 43.5, df = 1, *p* < 0.001; Figure 7). The tail-up position was observed in higher proportions in cat–human interactions than in cat–cat interactions (t = 11.27, df = ∞, *p* < 0.001; Figure 7).

## 4. Discussion

Only 126 dyads out of 406 possible ones in the 29-cat sample were involved in the interactions which illustrates the non-social though facultative gregarious character of the species [22,23,24,33,41]. Possibly because of this unique character and its large range of intraspecific tolerance, the domestic cat, *Felis catus*, is an expressive species. Here we combined the visual configurations described by Leyhausen [23], considering ears and the tail, regardless of the opening of the mouth, generally associated with vocalizations (what we would have referred to as “multimodal configurations”). We showed that, in cat–cat interactions, the position of the ears was the most important features in influencing the interaction.

Kiley-Worthington [9] mentioned that tail position in cats is more a matter of tonus than that of communication. We did not consider here the different and complex movements of the tail of the cat.

Our results only partially support Cameron-Beaumont’s assertions that the tail-up display is an affiliative signal [33,35]. She considered only the positions of the tail regardless of other visual signals such as the position of the ears [35]. In addition, she analyzed separately the initiator and the recipient cat for each interaction [35]. Cafazzo and Natoli [33], who also only considered the position of the tail, showed that the tail up was displayed more by cats who received the most aggression. In addition, these cats also were the youngest in the group (only one cat was juvenile, and was the only intact individual of the colony). Cafazzo and Natoli [33] proposed that the tail-up display was a friendly signal, functioning as a signal to inhibit aggression.

Kiley-Worthington [9], though dealing with the possible communicative function of tail postures, mentioned the ears and tail (what we referred here to as the “tail–ears configuration”) as important for communicative value, in cats. Our analysis of tail–ears configurations shows clearly that the position of the ears is more important than tail position for both cats when they interact. It is worth noting that Leyhausen showed only one tail up position among 16 configurations he presented for cat–cat interactions [23]. The position of ears is an important visual signal and is part of the facial configuration in cats as well as in other domestic species such as dogs and horses. As interactions include face-to-face contact, it is not surprising that the relative positions of ears are predictive of the outcomes of interactions. Ear postures likely convey a continuum of motivation along the three principal dimensions of interactive behavior: affiliation, defense, and threat [23]. Many mammals possess a rich musculature to move their ears [42]. Postures of ears have been investigated in some domestic species, such as horses [21], sheep [43], and dogs [20], in order to assess communicative value or more general expression of emotions. The position of the ears has been shown to be the more important component of the configuration than the position of the tail although the tail-up display is rather conspicuous. In contrast to what was observed in sheep [43], in cats, as in dogs and horses, the movements of ears are symmetrical within dyads. Only the erect ear position in both cats indicated a positive emotion leading to a positive outcome of the interaction.

### 4.1. Welfare Issues 

We showed that visual configurations used by cats when interacting with other cats or with humans differed greatly: in cat–cat interactions, the position of the ears appeared to be the most significant feature and when approaching humans, usually only one visual configuration is used, featuring the tail up. Correia-Caeiro et al. [44], dealing with dog–humans interactions, made the important point that what is important for one species might not be so for another one, especially when very phylogenetically distant. 

Ekman’s FACS [14] inspired recent research which explored fine details in the “visual face configuration” [28,30,31]. The results of these experimental studies were intended to perceive signs of pain or stress in cats. In terms of welfare it is of paramount importance, though it seems unlikely that cats, when interacting with other cats, were processing some or all the fine details of facial configurations revealed in these studies. As far as the position of the tail is concerned, Cafazzo and Natoli [33] proposed that dominant cats induced the tail-up approach from subordinate ones. Transposed to cats’ approaches to humans, one may hypothesize that cats consider humans as dominant to them. The question of dominance either in social species, such as primates [45], or in interspecific dog–human interactions has been criticized [26,46]. Looking at hierarchies built on aggression, they are often correlated to some other factors, such as sex, weight, or age [33,47]. Instead of considering humans as dominant to them, we propose that cats consider humans as their caregiver. When viewed in this way, the manner in which they approach humans is similar to the way kittens approach and greet their mothers, prior to suckling. Humans are very aware that tail-up approaches are friendly as they are generally followed by rubbing. Humans are also aware of other infantile behaviors that cats direct toward them: purring, kneading, suckling, gently rolling on the back, and even suckling (see [48]). Being aware that a tail-up approach with ears non-erect may have a different meaning than when ears are erect might be a way to better process the information given by cats. Salient features in expression of emotion have been selected during evolution, through different processes, to be the least ambiguous as possible and induce specific responses (see [4]). This is true for intraspecific interactions. In interspecific interactions, each species has to adapt and adjust to the communicative repertoire of the other species. Humans in particular may learn through associative learning the meaning of the signals given by their pets [44] Ear or tail positions are easily perceived by humans and interpreted. Artificial selection in cats has been less active than that in dogs [49]. In dogs, artificial selection has produced a huge variety of phenotypes, and as Bradshaw and Nott [50] noted, drooping ears and docked tails induce an important change in communicative ability from dogs which have a physical conformation more similar to wolves. Consequently, a significant number of cat breeds keep their ears and tail fully developed and easily visible, and therefore keeping their important capacity of expression. A further aspect of learning for humans, especially in presence of domestic animals, is, for example, to look at holistic configurations instead of focusing on only one feature, even if it is conspicuous (for examples of use of bimodal configurations, see [51]). 

### 4.2. Limitations

Although our study focused on a methodological point related to communication, many more aspects of group living in cats might having been studied. Video recordings were not used in this study. This surely should have been done, provided this study was not only part of a larger study about cat behavior living in a semi-free range group. As cats were all identified, the different ears–tail combinations could have been sorted out at a dyadic level and the effects of sex and age possibly explored. As the twenty-nine adult cats involved a certain number of males and females, visual configurations might have been analyzed at a male–male, female–female, or female–male level to better explore the function of these visual configurations. However, due to the low frequency of interactions and the low proportion of dyads interacting in regard to all possible dyads (28%), a much larger number of data should have been obtained. 

## 5. Conclusions

We showed that, in cat dyadic interactions, the position of the ears of both protagonists, similar or different, is a good predictor of the outcome of these interactions. Cats seemed to pay more attention to the head–face components of the visual configuration that also includes the position of the tail. 

Analyzing visual configurations in domestic species, such as cats, has a twofold importance: 1. to provide a better understanding of communicative behavior in cats, and 2. scientific results should be used to instruct humans and therefore participate in the preservation of the cat–human bond as well as promote the well-being of cats.

## Data Availability

The data presented in this study are available on request from the corresponding author.
